# Cellulose-functionalized magnetic nanoparticle-mediated isolation of novel cellulolytic bacteria

**DOI:** 10.1128/aem.00788-25

**Published:** 2025-08-29

**Authors:** Jieyi Zheng, Lei Xing, Nan Zheng, Jiaqi Wang, Shengguo Zhao

**Affiliations:** 1 State Key Laboratory of Animal Nutrition and Feeding, Institute of Animal Sciences, Chinese Academy of Agricultural Sciences 243826, , Beijing, China; Washington University in St. Louis, St. Louis, Missouri, USA

**Keywords:** rumen, magnetic nanoparticles, cellulolytic bacteria, isolation

## Abstract

The cellulolytic bacteria are crucial for applications in animal feeds, food processing, and bioenergy production. However, isolation of these bacteria from the microbial community has been challenging due to limitations in current methodologies. This study introduces a novel cellulose-functionalized magnetic nanoparticle-mediated isolation technique designed to enhance the recovery of cellulolytic bacteria. We hypothesized that bacteria could adhere to magnetic nanoparticles coated with cellulose, allowing selective isolation of cellulolytic strains through their cellulase activity-induced magnetic loss. The magnetic nanocomposite was synthesized and functionalized with cellulose (magnetic nanoparticle-cellulose composite [MNPC]). The anaerobic *Streptococcus bovis* was used to evaluate the bacteria capture efficiency of MNPCs. A mixture of *Cellulomonas flavigena* (a cellulolytic bacterium) and *S. bovis* (a non-cellulolytic bacterium) was used to evaluate the cellulolytic bacteria isolation specificity of the method. This method was also applied to isolate the novel ruminal cellulolytic bacteria. The results showed that the magnetic nanoparticle had an average size of 20.00 nm and superparamagnetism. MNPC achieved a bacterial capture efficiency of 99% and a high specificity. The method successfully captured the known cellulolytic bacteria from the rumen of cattle, including *Fibrobacter*, *Ruminococcus flavefaciens*, and *Butyrivibrio*, indicating its reliability. Furthermore, a novel cellulolytic species from the family *Lachnospiraceae* was isolated from the rumen, with genome analysis revealing cellulolytic pathway genes encoding endoglucanase, β-glucosidase, and cellobiose phosphorylase. In conclusion, the cellulose-functionalized magnetic nanoparticle-mediated isolation method presents a promising approach for the efficient enrichment and isolation of active cellulolytic bacteria. This technique and the isolated strains have potential applications in enhancing animal productivity, bioenergy production, and the food industry.

**IMPORTANCE:**

Lignocellulosic biomass, as the primary component of cell walls in gramineous plants, is widely recognized as an ideal biofuel feedstock due to its sustainability and renewability. It can be digested and degraded by cellulolytic microorganisms, converting it into absorbable proteins and volatile fatty acids that provide energy for the organism. However, isolation of these bacteria from the microbial community has been challenging due to the limitations in current methodologies. In this work, we developed a new method, the cellulose-functionalized magnetic nanoparticle-mediated isolation technology, which can contribute to capture highly active cellulolytic bacteria. We isolated a novel unclassified cellulolytic strain of the *Lachnospiraceae* from the rumen of cattle. This methodology presents a promising approach for the efficient enrichment and isolation of active cellulolytic bacteria from the community.

## INTRODUCTION

Microorganisms in the natural environment are characterized by high diversity and are closely linked to host health as well as physiological metabolism and other functions (1). It is estimated that 81% and 25% of the world’s uncultured microbial genera and phyla, respectively, represent a significant proportion of the ecosystem (2). Uncultured microorganisms play an important role in the entire ecosphere and may possess functions such as novel biosynthetic pathways and unknown biochemical characteristics (3). However, many microorganisms still exist in uncultured environments, which is a great challenge for the isolation of uncultured microorganisms. There are various microbial isolation and culture methods, such as population culture (microbial coculture, *in situ* culture) (4, 5) and cell capture (single cell capture isolation, fluorescence-activated cell isolation techniques) (6, 7) with high-throughput isolation techniques (culture histology, microdroplet culture method, bioreactor culture method) (8–10), which can isolate several new or uncultivated microorganisms. However, these methods have the disadvantages of low isolation efficiency, complex operation, or expensive equipment, which leads to difficulties in isolating new specific, especially functional active microorganisms and limits the knowledge of decomposition mechanisms and regulatory mechanisms of functional strains.

Magnetic nanoparticles (MNPs) are a class of nanoparticles that can be manipulated using magnetic fields. MNPs have been widely used in ferrofluids (11), biomedicines (12), chemicals (13), environmental remediation (14), catalysts (15), and spintronics (16) due to their strong saturation magnetization and good biological compatibility (17). With the capability of remote control by magnetic field, MNPs introduce many possibilities for enrichment and isolation as a novel tool in microbiology. Zhang et al. pioneered a new MNP-mediated isolation (MMI) method to isolate and identify the active microbial cells, especially bacteria belonging to the order *Burkholderiales*, that perform *in situ* phenol degradation from coking plant wastewater (18). Therefore, MMI provides a new methodology for isolating active functional microbes from the community.

Biomass, an alternative source to fossil resources, offers a promising opportunity for renewable energy (19). Biomass, specifically lignocellulose, which is composed of plant cell walls from the grass family, is considered a sustainable and renewable feedstock for biofuels (20). Lignocellulosic biomass consists mainly of three different types of polymers, namely cellulose, hemicellulose, and lignin, and is the most abundant raw material on earth for producing biofuels (21). It is now well established that the microbiome plays a pivotal role in lignocellulose digestion. The rumen of ruminants provides a natural bioreactor for the microbial community that naturally and efficiently hydrolyzes cellulose and ferments the resulting sugars to products that are precursors to biofuels (22). Genomic and metagenomic sequencing of the rumen microbial community revealed that diverse cellulolytic microbes digest cellulosic substrates, which proves that the rumen is one of the most efficient bioreactors (22). Rumen bacteria are an important population for forage degradation, which was a natural treasure to explore new bacteria (23, 24). In the rumen, the typed isolated cellulolytic bacteria are from *Fibrobacter*, *Ruminococcus flavefaciens*, and *Butyrivibrio* (25, 26). However, these cellulolytic bacteria, isolated using the traditional Hungate tube or plating method, merely represent the tip of the iceberg. To solve this problem, this study established a magnetic nanoseparation method that can be used to capture and isolate active functional microorganisms effectively, which is highly important for the mining of uncultured microorganisms and the exploitation and utilization of resources.

## RESULTS

### Description of the MNPC-mediated isolation method

**Fig 1:**
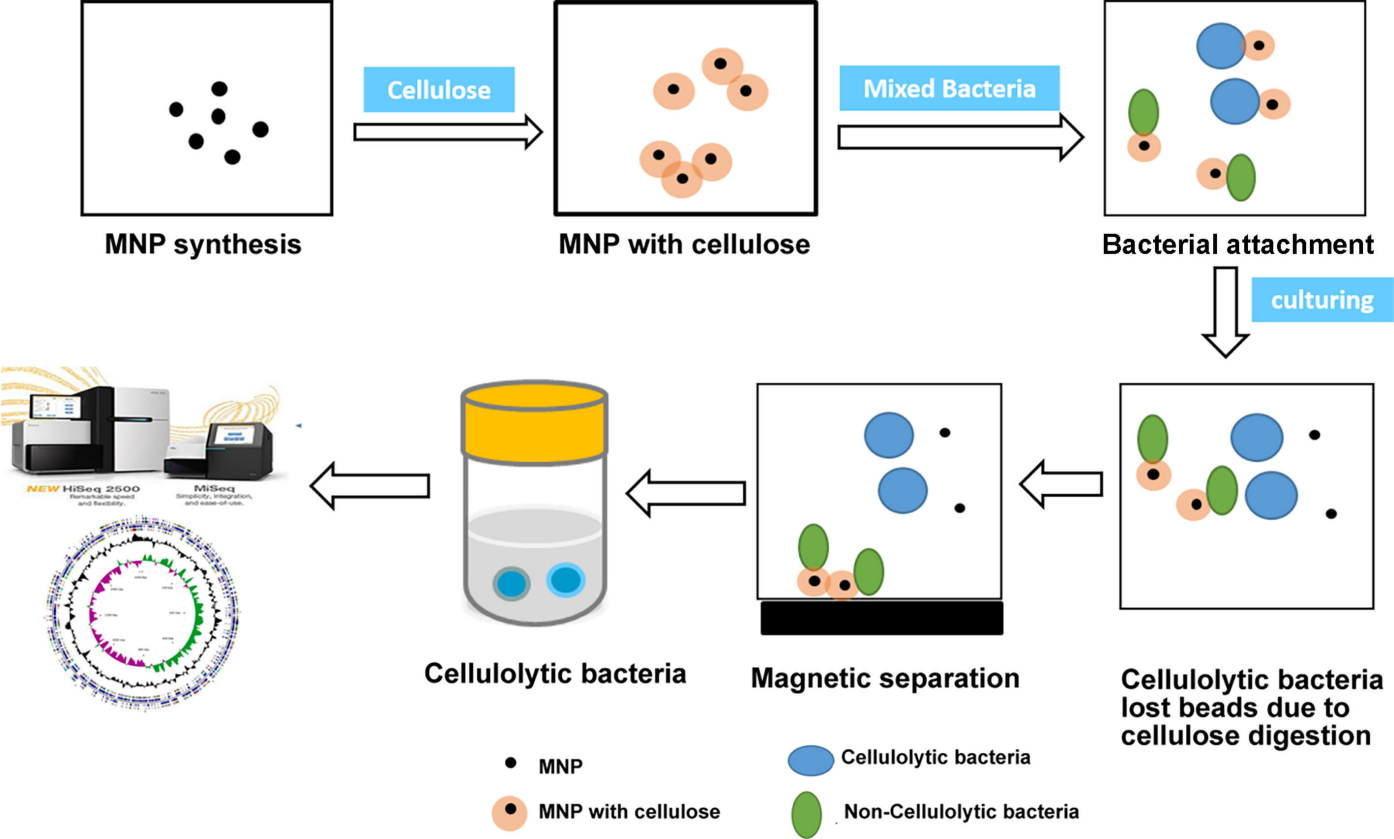
Schematic diagram of the enrichment and isolation of the cellulolytic bacteria by MNPC method. MNPs were first prepared using a co-precipitation method and subsequently coated with cellulose to obtain MNPC. The MNPC was then mixed with a microbial community. During incubation, the cellulolytic bacteria digested the cellulose on the MNPC and detached into the suspension, while the non-cellulolytic bacteria remained attached to the MNPC. The cellulolytic bacteria in the suspension were collected using permanent magnets. Finally, these isolated cellulolytic bacteria were subjected to genome sequencing or physiological analysis to identify the targeted strains.

The magnetic nanoparticle-cellulose composite (MNPC)-mediated isolation method is illustrated in Fig. 1. MNPs were first prepared using a co-precipitation method and subsequently coated with cellulose to obtain MNPC. The MNPC was then mixed with the microbial community. During incubation, the cellulolytic bacteria digested the cellulose on the MNPC and detached into the suspension, while the non-cellulolytic bacteria remained attached to the MNPC. The cellulolytic bacteria in the suspension were collected using permanent magnets. Finally, these isolated cellulolytic bacteria were subjected to genome sequencing or physiological analysis to identify the targeted strains.

### Characterization of MNP and MNPC

**Fig 2:**
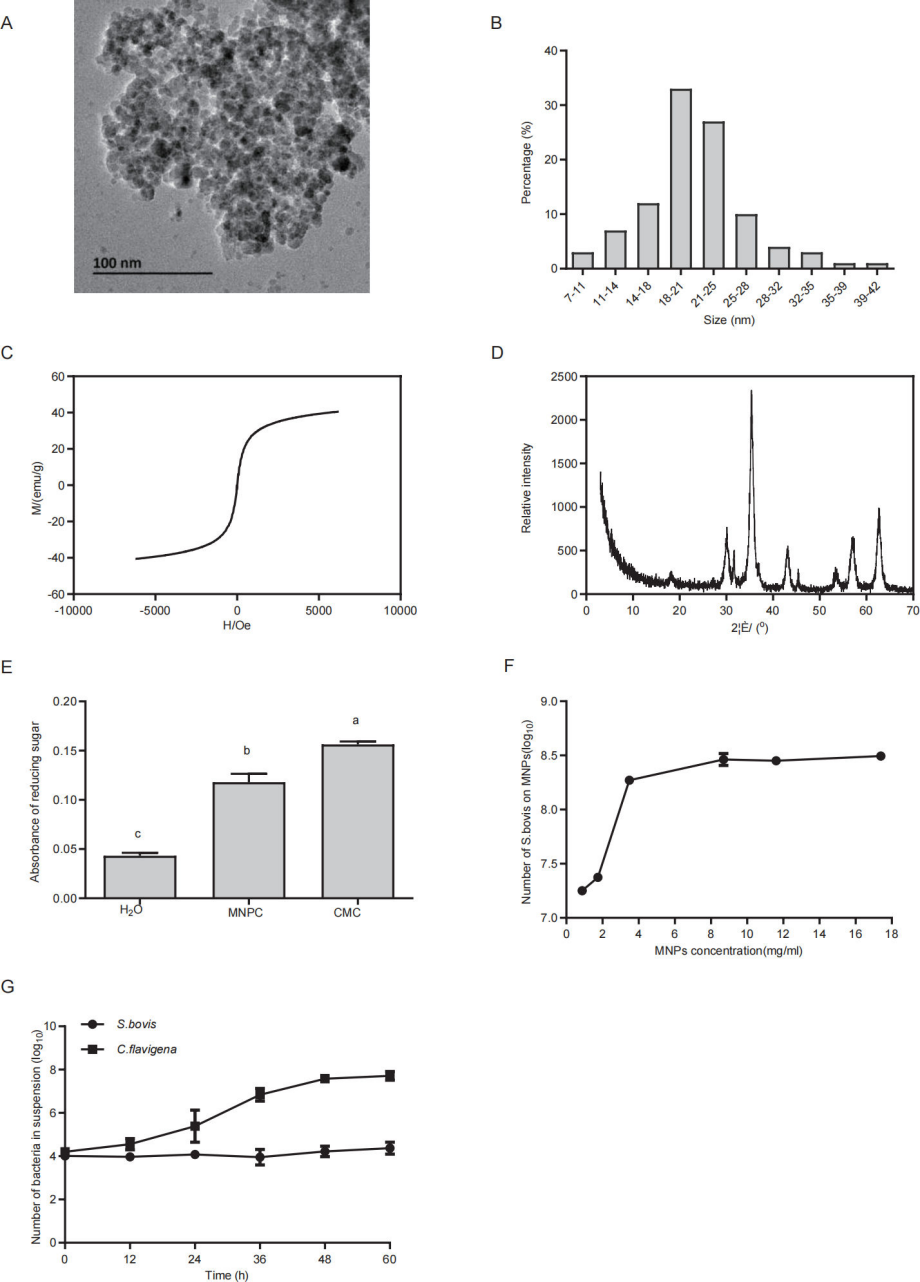
Characterization of MNP and MNPC or evaluation of the MNPC-mediated isolation method. (**A** through **E**) Characterization of MNP and MNPC. (**A**) TEM image of MNP. (**B**) MNP size distribution diagrams by Nano Measurer 1.2 software. (**C**) The magnetization curve of MNP. (**D**) The XRD pattern of raw MNPs to confirm the material type. (**E**) Efficiency of cellulose surface modification. (**F** and **G**) Evaluation of the MNPC-mediated isolation method. (**F**) The capture efficiency of MNPC against the bacteria. (**G**) The assessment of the isolation specificity of this approach. Data are presented as the mean percent ± SD. Negative control (ddH_2_O, *n* = 3), MNPC (*n* = 3), and positive control (carboxymethyl cellulose sodium, *n* = 3). a, b, c Different letters for different treatments indicate statistically significant differences (*P* < 0.05). The error bars represent one standard error of mean.

Transmission electron microscopy (TEM, H-9000NAR, Hitachi Limited) revealed that the synthesized MNPs were spherical with a size distribution of 20 ± 5.84 nm (mean ± SD) (Fig. 2A and B). The magnetization curves exhibited an S-shaped behavior, with a saturation magnetization of 43.4 emu/g (Fig. 2C). X-ray diffraction (XRD, Rigaku D/max-rA, Rigaku Corporation) analysis identified the diffraction peaks of MNPs at 2θ = 30.2°, 35.4°, 43.2°, 53.6°, 57.1°, and 62.7°, corresponding to the (220), (311), (400), (422), (511), and (440) lattice planes (Fig. 2D). The production of reducing sugars from MNPC confirmed successful cellulose functionalization (Fig. 2E).

### Evaluation of the MNPC-mediated isolation method

The capture efficiency of MNPC for bacteria, *Streptococcus bovis* (strain CGMCC 1.1624), is illustrated in Fig. 2F. As the concentration of MNPC increased, the number of bacteria bound to MNPC also increased. At an MNPC concentration of 8.70 mg/mL, saturation was achieved, and the maximum number of *S. bovis* reached 8.46 log_10_ CFU/mL. Therefore, the optimal concentration of MNPC was determined to be 8.70 mg/mL, with a capture efficiency of 99%. Figure 2G shows the isolation specificity. The number of non-cellulolytic bacteria, such as *S. bovis*, remained low throughout the 60 h incubation period. In contrast, the number of cellulolytic bacteria, such as *Cellulomonas flavigena* (strain ACCC 11055), increased from 12 h and stabilized at 48 h, rising from 4.21 log_10_ CFU/mL to 7.71 log_10_ CFU/mL. *S. bovis* showed minimal cellulose metabolism, evidenced by its low number in the suspension at 60 h.

### Changes in rumen bacteria incubated with MNPC

**Fig 3:**
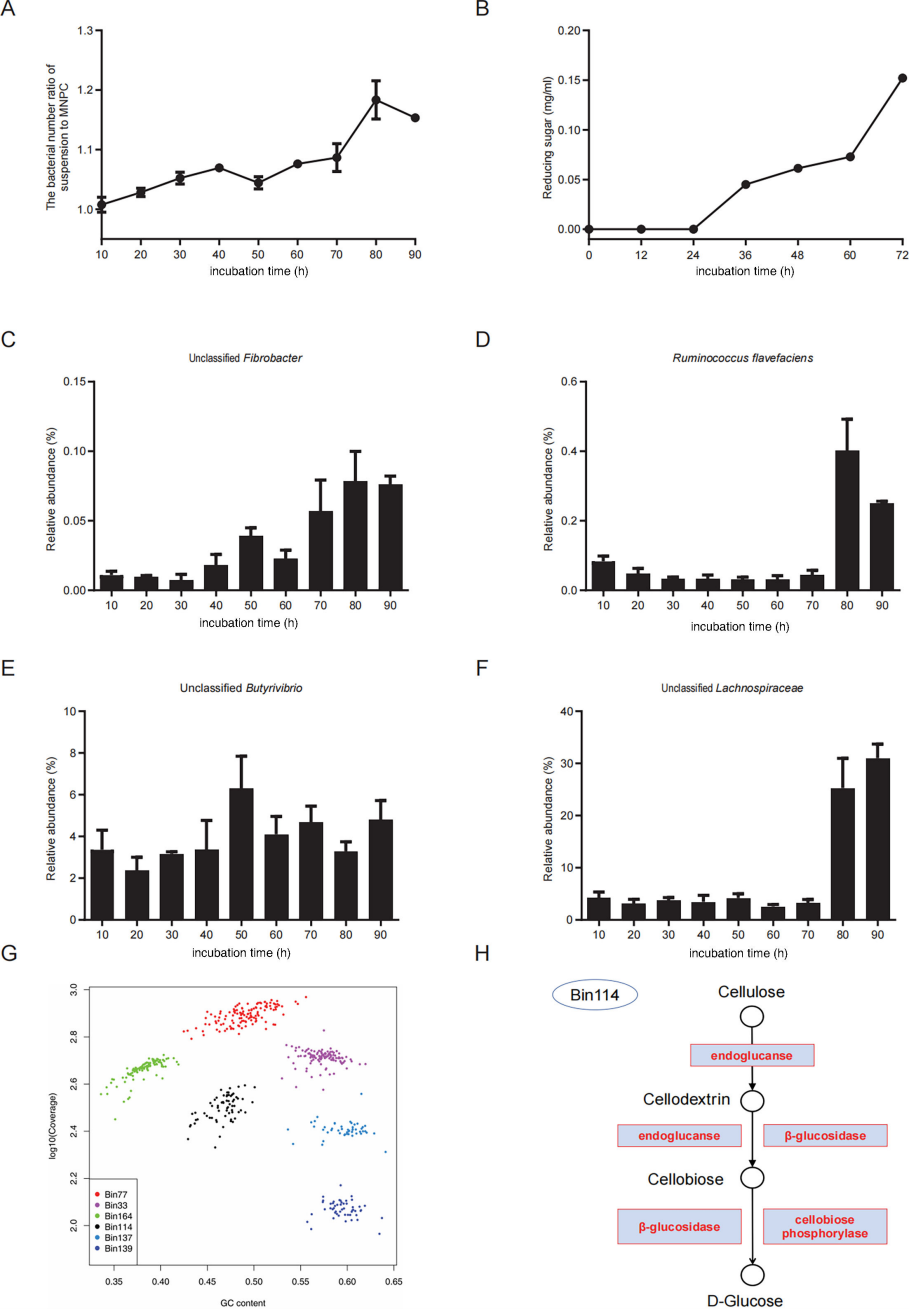
MNPC-mediated changes in isolated rumen bacteria and analysis of their genomes and cellulase genes. (**A** and **B**) 16S changes in rumen bacteria during 90 h. (**A**) The number of bacteria in the suspension (log_10_). (**B**) The number of bacteria in the MNPC (log_10_). (**C** through **F**) The relative abundance of typical cellulolytic bacteria determined by 16S rRNA. (**C**) Changes in relative abundance of unclassified *Fibrobacter* during 90 h of continuous culture. (**D**) Changes in relative abundance of *R. flavefaciens* during 90 h of continuous culture. (**E**) Changes in relative abundance of unclassified *Butyrivibrio* during 90 h of continuous culture. (**F**) Changes in relative abundance of unclassified *Lachnospiraceae* during 90 h of continuous culture. The error bars represent one standard error of mean. (**G**) GC-coverage plots for seven bins (plot symbols are scaled by contig length). (**H**) Enzymes involved in the pathway of degrading cellulose to glucose in Bin 114. The error bars represent one standard error of mean.

The ratio of bacteria in the suspension to those bound to MNPC increased gradually, peaking at 80 h (Fig. 3A). To identify the optimal time for isolating cellulolytic bacteria, the cellulose degradation dynamics were monitored. The content of reducing sugars increased progressively after 24 h, continuing to rise until 72 h (Fig. 3B).

### Changes in the relative abundance of typical rumen bacteria

The analysis of 16S rRNA gene sequencing data revealed changes in the relative abundance of typical rumen cellulolytic bacteria (Fig. 3C through F). In the suspension, the relative abundances of unclassified *Fibrobacter*, *R. flavefaciens*, and unclassified *Butyrivibrio* increased from 0.01, 0.08, and 3.37% to 0.08, 1.00, and 4.82%, respectively, from 10 to 90 h of incubation (Fig. 3C through E). Additionally, an unclassified bacterium from *Lachnospiraceae*, which ultimately had a high relative abundance of 31.00%, exhibited a growth trend similar to that of the typed cellulolytic bacteria, indicating the potential presence of novel cellulolytic bacteria (Fig. 3F).

### Genomic and cellulase gene analysis of unclassified *Lachnospiraceae*

Metagenomic sequencing of MNPC-mediated enriched cellulolytic bacteria yielded 189,055,976 high-quality paired-end reads, representing 28 Gbp of sequences. *De novo* assembly with SPAdes resulted in 1,502,637 contigs of length >200 nt. Binning analysis identified 168 genome bins, with 43 high-quality bins showing >80% completeness and <5% contamination. The top six abundant bins (>6.5 reads per kilobase per million mapped reads [RPKM]) are shown in Fig. 3G, with Bin114, an unclassified *Lachnospiraceae*, demonstrating high abundance and quality (supporting information: Table S1). Functional annotation of Bin114 identified genes encoding endoglucanase, β-glucosidase, and cellobiose phosphorylase, which are essential enzymes involved in cellulose degradation (Fig. 3H). Additionally, B114 contains genes associated with carbohydrate metabolism, including glycosyl hydrolase and glycosyl transferase families. Genes encoding enzymes such as multi-copper polyphenol oxidoreductase laccase, α-galactosidase, β-galactosidase, arabinogalactan endo-1,4-β-galactosidase, α-l-rhamnosidase, and α-amylase were also detected. These enzymes contribute to the degradation of lignin, hemicellulose, and starch.

## DISCUSSION

Uncultured bacteria play crucial roles in cellulose biomass degradation *in situ* (18, 27–29), yet their isolation remains a significant challenge. MNPs have emerged as a promising tool for bacterial isolation due to their superparamagnetism and biological affinity, which facilitate rapid cell adhesion and separation (30–34). In this study, we introduced a novel method using MNPCs to enrich and isolate the cellulolytic bacteria from the complex microbial communities, specifically from the rumen of cattle. The cellulose-functionalization of MNPCs allowed the cellulolytic bacteria to adhere to and degrade the cellulose on MNPCs, leading to the loss of magnetic attraction and subsequent detachment. In contrast, the non-cellulolytic bacteria remained attached to the MNPCs and could be removed using a magnet (Fig. 1).

Co-precipitation is a tractable and efficient method to control the size and composition of MNP versus conventional approaches (35). Magnets can effectively capture MNP immediately. Meanwhile, magnetization curves were “S” shaped and the highest saturation magnetization was 43.4 emu/g. Similar results were also observed in a previous study (36) which demonstrated that the magnetization was fit for capture. We could observe the characteristic peaks consistent with the Fe_3_O_4_ peaks, and no impurity peaks were observed. Most of the particle sizes of MNPs ranged from 14 to 28 nm. The diameter is close to that of previous reports (37, 38). We used the 3,5-dinitrosalicylic acid (DNS) method to qualitatively estimate the efficiency of cellulose-Fe_3_O_4_ synthesis. Results showed that the absorbance of water (negative control) was 0.04 and MNPC (treatment group) was 0.10, which illustrates that MNPs were effectively packaged by cellulose. During our experimental period, some key points need to be improved. First, the whole preparation process should be performed under an N_2_ atmosphere, which can prevent O_2_ from oxidizing iron dichloride into ferric trichloride. Second, during synthesis, we used ultrasonication to replace the stirrer. Compared with other technologies, such as the mechanical ball grinding method (39), hydrothermal method (40), and micro-emulsion method (41), ultrasonic cavitation is easier to eliminate local unevenness, enhances the reaction speed, and stimulates the production of a new phase.

MNPCs were able to effectively attach to bacteria based on electrostatic adsorption. The concentration of MNPC was the key factor for effective bacteria capture. When the concentration of MNP was above 8.70 mg/mL, the bacteria capture efficiency was close to saturation. To ensure that all bacteria could be captured, 8.70 mg/mL was chosen as the optimum MNP concentration for subsequent analyses. Cultivated in anaerobic dilution medium, *S. bovis* could not metabolize cellulose, remaining on the surface of MNPC. *C. flavigena* was released from the MNPC due to cellulose consumption. *S. bovis* was nearly still, while *C. flavigena* kept growing until 48 h. Thus, *C. flavigena* was enriched and isolated in the supernatant from the artificial microbiota.

The gradual increase in the bacterial number ratio of suspension to MNPC revealed that the bacteria in the suspension were constantly enhanced. In detail, the cellulolytic bacteria were growing, and the non-cellulolytic bacteria could not utilize cellulose down to death. The utilization of cellulose remaining in the MNPC by cellulolytic bacteria might account for the result. The concentration of reducing sugar did not increase until 24 h. Therefore, the result provided reference evidence for us to confirm the time point to isolate cellulolytic bacteria in the rumen.

Traditionally, *Fibrobacter*, *R. flavefaciens*, and *Butyrivibrio* have been recognized as key anaerobic cellulolytic bacteria in the rumen of cattle (42–46). The application of MNPCs led to a significant increase in the capture of these typical cellulolytic bacteria, while non-cellulolytic bacteria, such as *Prevotella*, were significantly reduced. This demonstrates the efficiency of the MNPC-based isolation method. Notably, we also observed an increase in an uncultured species from the *Lachnospiraceae* family. Given that cellulose served as the sole carbon source in this system, this population surge strongly suggests previously unrecognized cellulose-degrading capacity in this bacterium. Genome binning of this uncultured *Lachnospiraceae* species revealed genes encoding endoglucanase, β-glucosidase, and cellobiose phosphorylase, all critical enzymes for cellulose metabolism. Cellulose degradation is initiated by glucanases cleaving β-1,4-glycosidic bonds to yield cellodextrins/cellobiose, followed by β-glucosidase-mediated hydrolysis to glucose. Cellobiose phosphorylase alternatively catalyzes phosphorolysis of cellobiose into α-d-glucose-1-phosphate and d-glucose. This enzyme occurs in anaerobic cellulolytic bacteria (e.g., *Ruminococcus albus*, *Clostridium stercorarium*) (47), indicating that the uncultured *Lachnospiraceae* represents a novel cellulolytic taxon in the rumen. Furthermore, these uncultured *Lachnospiraceae* encode multiple enzymes simultaneously, including multi-copper polyphenol oxidoreductase laccase, α-galactosidase, β-galactosidase, arabinogalactan endo-1,4-β-galactosidase, and α-l-rhamnosidase. Laccase, a multicopper oxidase found in bacteria, fungi, and other microorganisms within animal hosts, functions in lignin degradation (48). Hemicellulose primarily consists of xylans (with xylose forming the backbone) bearing various substituent groups on their side chains; consequently, its enzymatic hydrolysis requires additional enzymes such as α-galactosidase, β-galactosidase, arabinogalactan endo-1,4-β-galactosidase, and α-l-rhamnosidase to hydrolyze these side-chain sugar residues (49–51). Notably, the uncultured *Lachnospiraceae* also possess α-amylase, which efficiently catalyzes the hydrolysis of starch molecules into readily absorbable small molecules such as maltose, oligosaccharides, and dextrins.

*Lachnospiraceae* was recognized as the main cellulolytic bacteria in the geese (52). The *Lachnospiraceae* family exhibits significant cellulolytic capabilities, with representative genera including *Butyrivibrio* and *Lachnospira*. Specifically, *Butyrivibrio* serves as a core cellulolytic genus in the rumen, degrading cellulose and xylan through the secretion of enzymes such as cellulases and xylanases. It efficiently breaks down various hemicelluloses, pectin, and starch to produce glucose and butyrate, providing essential energy sources for the host (53, 54). In contrast, *Lachnospira* specializes in degrading soluble fibers like pectin and arabinan, yielding primarily acetate and ethanol as metabolic end products (55). In gut environments, the ability to degrade cellulose and hemicellulose components of plant material enables members of the *Lachnospiraceae* to decompose substrates that are indigestible by the host (56). The high cellulolytic activity of this family underscores its importance in cellulose processing (57). This study identifies the uncultured *Lachnospiraceae* as a putative novel genus. In-depth analysis reveals that, compared with other rumen cellulolytic bacteria, the uncultured *Lachnospiraceae* not only possess the capability to degrade cellulose, hemicellulose, and starch but also harbor genes encoding lignin-degrading enzymes, suggesting that they may have metabolic characteristics different from other rumen cellulolytic bacteria.

This method employs MNPs coated with cellulose on their surface, enabling specific enrichment of cellulolytic bacteria but not other strains. However, this represents a versatile strategy. By altering the surface-coated polymer (e.g., pectin or starch), functionalized MNPs can be synthesized to enrich bacteria capable of degrading specific polymers, such as pectin- or starch-degrading bacteria. It should be noted that this approach achieves enrichment of functional strains but cannot accomplish single-cell isolation. Subsequent isolation of individual strains requires a combination of dilution spread plating. Compared to conventional separation techniques that merely add cellulose to the culture medium yet fail to separate degrading and non-degrading communities, this method employs cellulose-coated MNPs to achieve selective separation of cellulolytic consortia. It specifically enriches functional microbiota with cellulolytic activity, significantly enhancing subsequent single-strain isolation efficiency while shortening the enrichment cycle relative to traditional approaches. Future research should focus on isolating this uncultured *Lachnospiraceae* strain using genome-guided methodologies to further investigate its biological role in fiber digestion within the rumen. While our study concentrated on identifying and validating new cellulolytic bacteria, the data sets generated offer a valuable resource for discovering additional genes and enzymes involved in cellulose degradation. In summary, we have developed a novel method using MNPCs for the efficient enrichment and isolation of active cellulolytic bacteria from complex microbial communities. The successful application of this method not only enhances our capability to isolate specific functional bacteria but also opens up new avenues for research into microbial communities and their roles in biomass degradation.

## MATERIALS AND METHODS

### Bacterial strains and culture medium

*C. flavigena* (strain ACCC 11055) was obtained from the Agricultural Culture Collection of China (Beijing, China) and *S. bovis* (strain CGMCC 1.1624) was obtained from the China General Microbiological Culture Collection Center (Beijing, China).

The anaerobic bacterial culture medium contained the following components: 2.0 g tryptone, 0.5 g yeast extract, 0.5 g glucose, 0.5 g cellobiose, 38 mL solution 2, 38 mL solution 3, 3.1 mL volatile fatty acid, 1 mL hemin (0.5 mg/mL), 5 g NaHCO_3_, 1 mg resazurin, and distilled H_2_O added to bring the final volume to 1,000 mL. Solution 2 (in 1000 mL of distilled water) contained 6.0 g K_2_HPO_4_. Solution 3 (in 1,000 mL of distilled water) contained 1.21 g CaCl_2_, 6.0 g KH_2_PO_4_, 12.0 g NaCl, 6.0 g (NH_4_)2SO_4_, and 12.5 g MgSO_4_·7H_2_O. Volatile fatty acid mix contained 17 mL acetic acid, 6 mL propionic acid, 4 mL n-butyric acid, and 1 mL each of n-valeric, isovaleric, isobutyric, and 2-methylbutyric acid. The medium was degassed with 100% CO_2_ for 2 h, adjusted to pH 6.8, and supplemented with 0.25 g l-cysteine HCl-H_2_O. It was then transferred to an anaerobic chamber (PLAS-LAB 855-AC & 855-ACB) immediately, dispensed into Hungate tubes, and autoclaved at 121°C for 20 min. The anaerobic dilution solution (in 500 mL of distilled water) containing 19 mL solution 2, 19 mL solution 3, 2.5 g NaHCO_3_, 0.5 mg resazurin, and 1.25 g l-cysteine HCl-H_2_O was anaerobically prepared similarly for specific applications.

### Synthesis and characterization of MNP and MNPC

MNPs were synthesized via the co-precipitation method (18, 58). Briefly, 1 mL of a solution containing 1 M FeCl₂ and 2 M FeCl₃ was added dropwise to 25 mL of 1.0 M ammonia solution under continuous stirring and ultrasonication (40  kHz, 75W). The reaction was allowed to proceed for 30 min until a dark iron oxide precipitate formed. The synthesized MNP precipitate was separated using a permanent magnet. The supernatant was discarded, and the precipitate was washed repetitively with an equal volume of ultrapure water until pH 7.0. At room temperature, add 5 mL of MNP to 40 mL of a 20 mg/mL cellulose suspension (prepared with 14 g NaOH and 24 g urea in 162 mL H_2_O). The MNP coated with cellulose was continuously stirred and shaken for 25 min. Subsequently, they were captured with a magnet for 5 min and washed with ultrapure water until pH 7.0, ultimately yielding cellulose-functionalized MNPs. The morphology of MNPs was characterized by TEM. XRD was used to identify the MNP composition. The magnetic property of MNP was analyzed by a vibrating sample magnetometer (VSM, SQUID-VSM, Quantum Design). To evaluate the cellulose content of MNPC, 3 mL MNPC, water (negative control), and carboxymethyl cellulose (positive control, 5 mg/mL) were mixed with 1 mL of cellulase solution (1 mg/mL), respectively, and incubated at 40°C for 30 min. The produced reduced sugar content was detected by the DNS method (59).

### Evaluation of capture efficiency and isolation specificity of MNPC

To determine the capture efficiency of MNPC, 0.5 mL *S*. *bovis* (1.0 × 10^8^ CFU/mL) culture was mixed with 0.5 mL of MNPC at various concentrations (17.4, 11.6, 8.7, 3.48, 1.74, and 0.87 mg/mL). After capturing with a magnet for 5 min and discarding supernatants, the rest MNPC was used for total bacterial quantification by qPCR using 16S rRNA gene primers as described previously (60). To assess the isolation specificity of bacteria on MNPC, the 0.5 mL MNPC (17.4 mg/mL) was mixed with an equal volume of the mixture of *S. bovis* (1.0 × 10^8^ CFU/mL) and *C. flavigena* (1.0 × 10^8^ CFU/mL), and the suspension after magnet capturing was discarded and replaced with anaerobic bacterial culture medium. During incubation at 30°C, the suspension was sampled every 12 h until 60 h and used for DNA extraction and qPCR analysis using *S. bovis* (forward: 5′-AGAGTITGATC (C/A) TGGCT-3′) and (reverse: 5′-ATGATGGCAACTAACAATAGGGGT-3′) as described previously (61) and *C. flavigena* primers (forward: 5′-SCCGCAAGGCTAAAACYCAAAGAAA-3′, reverse: 5′-CAWRACSYGCTGGCAACATRGGACG-3′) designed by us with an annealing temperature of 58°C.

### Cellulose degradation of MNPC by ruminal mixed microbes

Ruminal contents were obtained from three Chinese Holstein dairy cows (550 ± 50 kg) fitted with ruminal fistulae. The basic ingredient composition and nutritional composition are shown in Table S2. Approximately 300 grams of rumen contents were collected from each dairy cow through a rumen fistula after morning feeding and mixed. Rumen contents were filtered with four layers of cheesecloth. The solid fraction was washed with anaerobic dilution solution three times to get the solid-associated microbes. The liquid- and solid-associated microbes were mixed together to make a rumen microbial mixture. The aliquots were dispensed into penicillin bottles containing 4 mL rumen microbial mixture, 4 mL anaerobic dilution solution, and 2 mL glycerol. The bottles were stored at −80°C for the following experiments. To identify the proper time point to enrich and isolate the cellulolytic bacteria, 0.5 mL rumen microbial mixture was mixed with 0.5 mL MNPC for 20 min and then incubated at 39°C for 72 h. At 0, 12, 24, 36, 48, 60, and 72 h, 1 mL of sample was collected and centrifuged at 10,000 g for 5 min, then the supernatant was collected and used for the detection of reduced sugar using the DNS method (59).

### Enrichment and 16S rRNA gene sequencing of cellulolytic bacteria by MNPC

The experiment was conducted in an anaerobic chamber (PLAS-LAB 855-AC and 855-ACB). The rumen microbial mixture was centrifuged at 10,000 × *g* at 4°C for 5 min and resuspended with anaerobic dilution solution. MNPC was mixed with resuspended microbes for 20 min and captured by a permanent magnet for 5 min. The supernatants were discarded and replaced with an equal volume of anaerobic dilution solution. The samples were incubated at 39°C for 90 h. The samples were collected every 10 h and were determined by qPCR using total bacterial primers (60) and bacterial 16S rRNA gene amplicon sequencing (62). Purified bacterial 16S rRNA gene amplicons were paired-end sequenced (2 × 350 bp) on an Illumina MiSeq platform (Illumina, San Diego, USA). Operational taxonomic units were clustered with 97% similarity using UPARSE in QIIME 1.9 (63). The taxonomy of each 16S rRNA gene sequence was analyzed by the RDP Classifier algorithm against the GreenGene database using a confidence threshold of 80% (64).

### Genome binning and cellulolytic genes screening

Shot-gun DNA sequencing from the suspension enriched from rumen bacteria samples at 90 h was performed. A library was prepared using a Nextera DNA Library Preparation Kit (Illumina, San Diego, CA, USA) according to the manufacturer’s protocol. The library was sequenced in paired-end mode (2 × 150 bp) on a single lane of an Illumina HiSeq 2000 System. Raw reads were trimmed using Trim Galore to get clean reads (65).

*De novo* assembly was performed to get contigs with the SPAdes Genome Assembler (v3.9.0) (66). To reconstruct draft genome bins, the contigs longer than 1,500 bp were clustered using MetaBat v0.32.4 (67). Genome bins were assessed in terms of their completeness and contamination using the CheckM program (68). Only those draft genome bins assessed as being >80% completeness and <5% contamination were retained. The GC content was calculated by BedTools (69). The relative abundance of genome bins was measured by mapping the clean reads against the contigs using Bowtie2 (70), and was defined as the RPKM value, which was calculated as the number of mapped reads per kilobase of draft genome bin per million clean reads. The taxonomy of each genome bin was analyzed by GTDB-Tk (71). Functional genes in each bin were predicted with MetaGeneMark (72), and functional annotated by BLASTP search against the eggNOG database (73). The bins with RPKM value of >6.5 were selected and screened for the cellulolytic-related genes by KEGG Mapper (74).

### Statistical analysis

Statistics were performed with the use of GraphPad Prism version 6.01 (GraphPad Software, Inc., San Diego, CA, USA). The data obtained from the study were subjected to statistical analysis using Tukey test and one-way analysis of variance using SPSS 17.0 at the 0.05 confidence level.

## Data Availability

All sequencing data have been deposited in the GSA under submission number CRA018929 at https://ngdc.cncb.ac.cn/gsa/browse/CRA018929 (metagenome sequencing) and the BioProject database under accession number PRJCA030006 at https://ngdc.cncb.ac.cn/bioproject/browse/PRJCA030006. All data analyzed during this study are included in this published article and its supplemental material.

## SUPPLEMENTAL MATERIAL

Supplemental tables aem.00788-25-s0001.xlsx Table S1, basic genome characteristics of the recovered genome bins; Table S2, diet composition and nutrient composition of basal diet in the experiment.

ASM does not own the copyrights to Supplemental Material that may be linked to, or accessed through, an article. The authors have granted ASM a non-exclusive, world-wide license to publish the Supplemental Material files. Please contact the corresponding author directly for reuse.
